# Clinical Utility of SARS-CoV-2 Serological Testing and Defining a Correlate of Protection

**DOI:** 10.3390/vaccines11111644

**Published:** 2023-10-26

**Authors:** Kimia Sobhani, Susan Cheng, Raquel A. Binder, Nicholas J. Mantis, James M. Crawford, Nkemakonam Okoye, Jonathan G. Braun, Sandy Joung, Minhao Wang, Gerard Lozanski, Christopher L. King, John D. Roback, Douglas A. Granger, Suresh B. Boppana, Amy B. Karger

**Affiliations:** 1 Department of Pathology and Laboratory Medicine, Cedars Sinai Medical Center, Los Angeles, CA 90048, USA; 2 Department of Cardiology, Smidt Heart Institute, Cedars Sinai Medical Center, Los Angeles, CA 90048, USA; susan.cheng@cshs.org (S.C.);; 3 Department of Medicine, University of Massachusetts Chan Medical School, Worcester, MA 01655, USA; 4 Wadsworth Center, New York State Department of Health, Albany, NY 12201, USA; 5 Department of Biomedical Sciences, School of Public Health, University at Albany, Albany, NY 12222, USA; 6 Department of Pathology and Laboratory Medicine, Donald and Barbara Zucker School of Medicine at Hofstra/Northwell, Hempstead, NY 11549, USA; 7 F. Widjaja Inflammatory Bowel Disease Institute, Cedars Sinai Medical Center, Los Angeles, CA 90048, USA; 8 Department of Pathology, The Ohio State University Wexner Medical Center, Columbus, OH 43210, USA; 9 Department of Pathology, Case Western Reserve University and Veterans Affairs Research Service, Cleveland, OH 44106, USA; 10 Department of Pathology and Laboratory Medicine, Emory University School of Medicine, Atlanta, GA 30322, USA; 11 Institute for Interdisciplinary Salivary Bioscience Research, University of California Irvine, Irvine, CA 92697, USA; 12 Department of Pediatrics and Department of Microbiology, Heersink School of Medicine, UAB, Birmingham, AL 35233, USA; 13 Department of Laboratory Medicine and Pathology, University of Minnesota, Minneapolis, MN 55455, USA; karge026@umn.edu

**Keywords:** SARS-CoV-2, serology, antibodies, clinical utility

## Abstract

Herein, we review established clinical use cases for SARS-CoV-2 antibody measures, which include diagnosis of recent prior infection, isolating high titer convalescent plasma, diagnosing multisystem inflammatory syndrome in children (MIS-C), and booster dosing in the immunosuppressed and other populations. We then address whether an antibody correlate of protection (CoP) for SARS-CoV-2 has been successfully defined with the following considerations: Antibody responses in the immunocompetent, vaccine type, variants, use of binding antibody tests vs. neutralization tests, and endpoint measures. In the transition from the COVID-19 pandemic to endemic, there has been much interest in defining an antibody CoP. Due to the high mutability of respiratory viruses and our current knowledge of SARS-CoV-2 variants defining a CoP for prevention of infection is unrealistic. However, a CoP may be defined for prevention of severe disease requiring hospitalization and/or death. Most SARS-CoV-2 CoP research has focused on neutralization measurements. However, there can be significant differences in neutralization test methods, and disparate responses to new variants depending on format. Furthermore, neutralization assays are often impractical for high throughput applications (e.g., assessing humoral immune response in populations or large cohorts). Nevertheless, CoP studies using neutralization measures are reviewed to determine where there is consensus. Alternatively, binding antibody tests could be used to define a CoP. Binding antibody assays tend to be highly automatable, high throughput, and therefore practical for large population applications. Again, we review studies for consensus on binding antibody responses to vaccines, focusing on standardized results. Binding antibodies directed against the S1 receptor binding domain (S1-RBD) of the viral spike protein can provide a practical, indirect measure of neutralization. Initially, a response for S1-RBD antibodies may be selected that reflects the peak response in immunocompetent populations and may serve as a target for booster dosing in the immunocompromised. From existing studies reporting peak S1-RBD responses in standardized units, an approximate range of 1372–2744 BAU/mL for mRNA and recombinant protein vaccines was extracted that could serve as an initial CoP target. This target would need to be confirmed and potentially adjusted for updated vaccines, and almost certainly for other vaccine formats (i.e., viral vector). Alternatively, a threshold or response could be defined based on outcomes over time (i.e., prevention of severe disease). We also discuss the precedent for clinical measurement of antibodies for vaccine-preventable diseases (e.g., hepatitis B). Lastly, cellular immunity is briefly addressed for its importance in the nature and durability of protection.

## 1. Background

Serological testing continues to play an essential role in clinical diagnosis and management of disease, with broad applications in infectious, inflammatory, and auto-immune illnesses. The utility of SARS-CoV-2 antibody measurement as part of research and surveillance efforts is established. Herein, we focus specifically on reviewing clinical use cases for measurement of antibody responses to SARS-CoV-2 infection and vaccination. As the pandemic evolved to an endemic there has emerged in the literature a set of circumscribed situations for which clinical practice guidelines may be developed—even while recognizing these may evolve further. To this end, issues related to reporting of antibody test results that can, in some instances, lead to misunderstandings by patients and clinicians are also reviewed. There has also been significant focus on determining an antibody measurement that is indicative of protective immunity, i.e., a correlate of protection (CoP). Most of this research has focused on neutralizing antibody measures, which is discussed. However, we also discuss the use of binding antibody measures for a CoP, particularly antibodies against the S1 receptor binding domain of the viral spike protein (S1-RBD). Binding antibody assays are more widely available for clinical use because they are more automated and have higher throughput than neutralization methods and are more readily standardized. By way of illustration, a review of precedence (e.g., Hepatitis B antibody measures) is provided. We proceed to summarize studies that attempt to define a SARS-CoV-2 CoP and/or define response to vaccines in immunocompetent adult populations. Lastly, a brief discussion regarding cellular immunity and its assessment for clinical purposes is provided.

### 1.1. Antigenic Specificity

The SARS-CoV-2 virus expresses four structural proteins of which spike (S) and nucleocapsid (N) are almost exclusively used as targets of antibody assays [1]. S and N proteins are targeted due to their strong immunogenicity resulting in measurable responses by both B and T cells. The S protein is composed of two subunits, S1 and S2. SARS-CoV-2 gains cell entry via RBD located on the S1 subunit, which binds to angiotensin-converting enzyme 2 (ACE2) receptor on the host cell, while S2 is responsible for triggering fusion of the virus to host cell membranes [2]. Since S1-RBD facilitates host cell entry this epitope is most considered in the literature when discussing measurement of antibodies that bind and prevent host cell entry, effectively “neutralizing” the virus, i.e., neutralizing antibodies (nAbs). This is also why S1-RBD became the primary initial target of SARS-CoV-2 vaccine development. However, Chi et al., demonstrated that nAbs can also bind to the N-terminal domain (NTD) of S1 [3]. Furthermore, it was observed that broadly neutralizing antibodies could be directed against the stem helix (SH) region and the fusion peptide (FP) region of the S2 subunit [4,5,6,7]. Nevertheless, most SARS-CoV-2 nAbs produced by the immune system target S1-RBD, and to a lesser extent S2 [3,4,8]. However, the epitope targets of S1 are more likely to succumb to selective pressure in the viral mutation process, enhancing the possibility of immune escape by variants. Conversely, neutralizing epitopes in the S2 subunit are more conserved than in S1. Therefore, nAbs targeting S2 epitopes have a greater likelihood of being broad-spectrum nAbs for SARS-CoV-2 variants. The latter feature becomes important both in designing new antibody therapeutics and potentially for updated versions of SARS-CoV-2 vaccines [9,10,11,12,13].

Overall, the SARS-CoV-2 S protein shares 76% sequence similarity with SARS-CoV-1 and only ~30% sequence identity with other seasonal β-coronaviruses (β-CoVs: e.g., OC43, HKU1) [14]. Among various CoVs, the N protein is the most abundant structural protein and immunogen in virus infected cells, with ~90% sequence identity between SARS-CoV-2 and SAR-CoV-1 [15,16]. However, like the S protein, the sequence identity of the N protein with other α and β-CoVs that cause the common cold is low at ~38–43% [17]; this indicates that the specificity of these two antigens for assessing an immune response to SARS-CoV-2 has the potential to be quite high; although S protein antibodies tend to persist longer than N protein antibodies [18]. Premkumar et al., demonstrated early in the pandemic that the S1-RBD antigen of SARS-CoV-2 was 100% specific for SARS-CoV-2 virus, as compared to SARS-CoV-1 and other common CoVs [19]. Relatedly, in 2020 the CDC set an antibody assay threshold for clinical specificity at ≥99.5% and this threshold has been met by most manufacturers of assays with Food and Drug Administration (FDA) emergency use authorization (EUA) [20,21].

### 1.2. The Precedent of an Antibody CoP for Other Viruses, and Lessons Learned

Thousands of published studies have examined or reviewed antibody responses to SARS-CoV-2 in various populations, both in response to infection and to vaccination. A PubMed search in early September 2023 using the phrase “SARS-CoV-2 antibody response” yielded 6425 results. However, when it comes to clinical utility of these antibody measurements one of the main goals is to link those responses to a measure of immune protection, i.e., define a CoP. We reviewed the literature for key studies that attempted to define a CoP based on neutralization and standardized binding antibody measures as tied to a specific endpoint (e.g., neutralization or prevention of severe disease). These are summarized in Table 1. Larger studies with defined endpoint measures are limited in the literature [22,23,24,25]. Therefore, studies that specifically measured peak binding antibody responses to vaccines in largely immunocompetent adult populations with results reported in standardized units (i.e., BAU/mL) are also summarized in Table 1**.** From this group of studies an anticipated peak IgG S1-RBD binding antibody response range post-vaccination can be determined and is discussed in Section 2.

Organizations such as the CDC maintain interim guidelines on antibody testing; at the time of this publication, these guidelines state that:

*“Antibody tests with very high sensitivity and specificity are preferred since they are more likely to exhibit high positive (probability that the person testing positive actually has antibodies) and negative predictive values (probability that the person testing negative actually does not have antibodies) when administered at least 3 weeks after the onset of illness.*

*Additional considerations when selecting an antibody test include:*

- *IgG levels appear to decrease more slowly over time than levels of other classes of antibody. Therefore, assays that measure total antibody or IgG could have higher sensitivity than IgM assays as more time passes since a person’s last infection.*
- *IgM antibody can persist for weeks to months following infection, though its persistence appears to be shorter than IgG’s; therefore, detection of IgM could suggest relatively recent infection.*
- *Detection of persistent antibodies varies by the test used.*

*FDA has issued an EUA for surrogate neutralization tests, which are qualitative binding assays that detect antibodies that block the interaction between the virus and the cellular virus receptor (ACE-2) without using cells or infectious virus. Plaque reduction neutralization assays are considered the gold standard for detection of neutralizing antibodies, but require cells, infectious virus, and are difficult to standardize [34].”*

These statements are congruent with using standardized binding antibody assays, particularly IgG, for most applications. The antigen target (e.g., S1, S2, N, etc.) is not specified in the excerpt because that depends on the reason for measurement. However, for vaccine response studies, detection of antibodies to N antigen is almost exclusively used as a retrospective marker for natural infection. This is because global vaccine development thus far, except for attenuated live-virus vaccines developed early on in China (i.e., CoronaVac and Sinovac), have elicited responses to S antigen using primarily mRNA, viral vector, and recombinant protein-based approaches. The live-attenuated approach has since demonstrated weaker responses than mRNA-based approaches [35].

The CDC stance also supports that neutralization assays are challenging to standardize and/or to employ. Of note, the gold standard for neutralization, plaque reduction neutralization tests (PRNTs), are not widely employed in the SARS-CoV-2 literature for the latter reason. We expand on interim and related statements by reviewing clinical use cases more closely (e.g., supplying booster shots and adequacy of humoral response) [36].

Evidence of immunity for most vaccine-preventable diseases (e.g., measles, mumps, rubella, chickenpox, poliomyelitis, diphtheria, pertussis, tetanus, hepatitis A, and even influenza) is a record of previous vaccination per a specified schedule or period. This is largely because the persistence of memory B and T cells, comprising the cellular immune response, is often long for most of these viruses. Key to persistence of immunity is a low mutation rate in important regions of these viruses. This is not because mutation is inherently unlikely but rather because of factors like limited host range and functional constraints on host target protein receptor binding [37,38,39]. This low, viable mutation rate does not hold for influenza viruses, particularly influenza A. Vaccines are redesigned yearly to account for the main viruses expected to make people sick from the four influenza A and B groups, and modeling does not get it right every year hence why effectiveness can vary year to year [40]. Even so, influenza vaccines prevent severe illness in a majority of the population if the recommended schedule is followed [41,42]. Nevertheless, serological testing is available for these diseases and is used to both help design vaccines and, in some cases, can be performed when vaccination records are inadequate or to assess response in immunocompromised individuals.

While not a good correlate to highly mutable respiratory viruses like SARS-CoV-2, Hepatitis B virus (HBV) is an example of a vaccine-preventable disease where serological testing is routinely utilized to assess immunity or vaccination status and for which consensus on an antibody correlate of protection (CoP) threshold has been achieved. Serological testing for immunity or exposure to HBV is mostly used for susceptible infants or immunocompromised individuals. Post-vaccination serology testing is not recommended after a routine vaccination series is completed in immunocompetent individuals. Per CDC guidance as of 2006, a CoP for antibodies to hepatitis B surface antigen (anti-HBs) at a level ≥10 mIU/mL has been established. It is recommended that anti-HBs is measured 1 to 3 months after completing a vaccination series (i.e., 2–3 doses in most individuals depending on vaccine type) [43,44]. However, achieving this antibody level does not provide absolute protection from infection in all individuals, but rather carriage of hepatitis B surface antigen or chronic infection [45].

It has been shown that immunocompetent individuals who initially achieve a level ≥ 10 mIU/mL after vaccination have virtually complete protection against acute or chronic infection even when anti-HBs later declines below this level. This protection is primarily due to cellular immunity and the persistence of memory B and T cells [46]. Anti-HBs has been observed to decline rapidly during the first year post-vaccination and less rapidly thereafter. Children who have completed a three-dose vaccination series exhibit low or undetectable anti-HBs 5–15 years later; and among adults completing the same series, anti-HBs declines below 10 mIU/mL in 30%–60% within 9–11 years [47,48]. Therefore, the persistence of HBV immunity over time is only correlated with the peak level of anti-HBs achieved immediately after primary vaccination.

While we are too early in the post-COVID-19 pandemic era to define the persistence of memory B and T cells to SARS-CoV-2, it has been previously observed that the memory T cell response to SARS-CoV-1 (a.k.a. SARS) persisted up to 11 years by Ng et al., and up to 17 years later by Le Bert et al. [49,50]. The effect of T cell response in mediating immunity to SARS-CoV-2, particularly for severe infection, has been studied in small groups and subpopulations. Essentially, T cells aid in lessening disease severity but are not sufficient alone to prevent severe disease without a humoral response. It was recently shown in a group of five patients with primary antibody deficiency (PAD) and lack of specific humoral response that all patients developed a robust T cell response to S and N antigens; however, their disease courses were severe or fatal and viral clearance was only observed after treatment with COVID-19 convalescent plasma and monoclonal antibody administration [51]. This further confirms the necessity of an adequate humoral response for viral clearance. Conversely, a review of studies of T cell responses to SARS-CoV-2 in patients with T cell lymphopenia but some degree of a humoral response, showed affected patients were not only symptomatic in their disease courses but tended to have more severe infections and morbidity [51]. This supports what may seem self-evident, both intact humoral and cellular immune responses are needed to prevent severe disease from SARS-CoV-2 infection.

While direct comparisons between HBV and SARS-CoV-2 serology certainly cannot be made due to significant differences between these viruses (e.g., chronic vs. acute disease courses, and stable vs. highly mutable viral structures), we can be guided by precedent. Much like with HBV, establishing immune protection based on the peak response achieved post-vaccination in immunocompetent cohorts may be a viable approach for SARS-CoV-2. However, unlike HBV, regularly updated vaccines to SARS-CoV-2 variants appear to be the way of the future. Protection conferred by HBV vaccination has been shown to last on the order of 30 years, and perhaps longer [52]. For immunocompromised individuals with higher risk of exposure to HBV, annual serological testing is recommended, and booster doses are required to maintain anti-HBs ≥ 10 mIU/mL. This scenario is where the clinical community may pursue somewhat routine clinical utilization of SARS-CoV-2 antibody measurements: assessing response in the immunocompromised and dosing them accordingly with boosters.

## 2. SARS-CoV-2 Antibody Levels in Immunocompetent Individuals

Enough time has passed from the onset of the pandemic that recent published reports have examined persistence of immune response beyond one year. Yang et al., has shown that neutralizing antibody responses persist in the majority (~86%) of mildly symptomatic convalescent individuals without subsequent vaccination up to 16 months after illness onset [53]. Persistent detection of neutralizing antibody responses in a majority (i.e., >85%) of convalescing individuals has also been demonstrated for more than a year by several groups [53,54,55].

**Figure 1:**
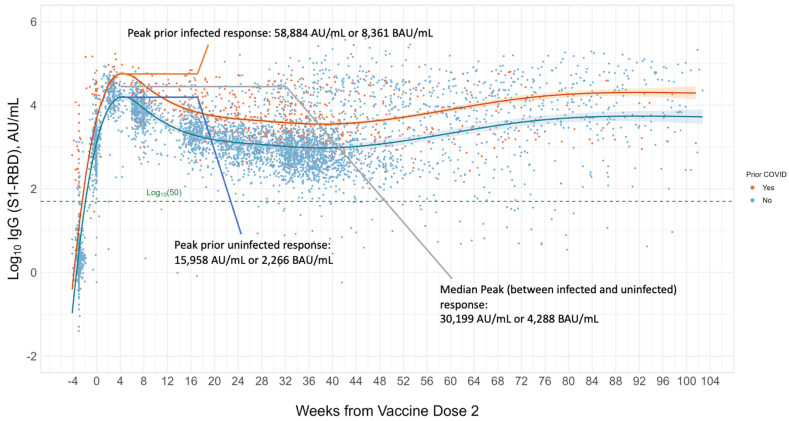
Longitudinal S1-RBD binding antibody response is shown for 1918 immunocompetent adult individuals across two years (104 weeks) from a large academic medical center cohort. The orange line represents those with prior infection at the time of vaccination and the blue line those with no prior infection. All completed a full regimen of primarily Pfizer BNT162b2 (73.5%), Moderna (20.5%), or other vaccines (6%). The median peak response (between prior infected and uninfected) is observed to be 30,199 AU/mL, corresponding to 4288 BAU/mL, and the peak prior uninfected response is 15,958 AU/mL or 2266 BAU/mL. The latter may better serve as a more conservative target for an expected immunocompetent response. A later rise in response is observed between 48 to 88 weeks, corresponding to booster doses and/or potential reinfection events. The dashed green line represents the threshold for positivity of the test employed. The majority of vaccinees remained above the positive threshold for the entire observation period.

A 10-month longitudinal cohort analysis of 843 healthcare workers at a large U.S. academic medical center, most of whom were immunocompetent, showed that 99.6% of patients maintained positive IgG S1-RBD levels (i.e., ≥50 AU/mL or 7.1 BAU/mL per Abbott Diagnostics) after completing a two-dose vaccination regimen of the Pfizer (BNT162b2) vaccine [56]. It is important to point out that exceeding the positive threshold for a given binding antibody assay is not the same as a “protective” immune response. One way to possibly define an adequate response in immunocompetent individuals is to determine the average or median S1-RBD range that is achieved ~4 weeks post final dose of vaccine. Such an approach would work well for both prior monovalent and current bivalent mRNA vaccines as well as recombinant protein vaccines on the market. This could also be applicable to adenovirus or viral vector vaccines targeting the same antigen and likely produce a lower target (e.g., Oxford/AstraZeneca AZD1222). The CoP targets may change or adapt if pan or universal vaccines based on more conserved protein structures of the virus (e.g., regions of the S2 subunit, and/or S1-NTD) are developed. In Figure 1, which was generated for this review by authors Cheng, Joung, Wang and Sobhani and expanded from previous work, we see average IgG S1-RBD responses plotted over two years (104 weeks) [56]. Responses were plotted over multiple blood draws for 1918 primarily immunocompetent adults at Cedars Sinai Medical Center who completed a full course of Pfizer (73.5%), Moderna (20.5%), and other (6%) vaccines. Of note, some individuals received additional booster doses, or experienced reinfections, hence the bump up in response between weeks 48 to 88. An Abbott Diagnostics IgG S1-RBD binding antibody assay was employed. We see that the peak median immune response between prior infected (orange line) and infection-naive at baseline (blue line) individuals was 30,199 AU/mL, or 4288 BAU/mL converted to standardized units. If we look at the average peak response of the infection-naive individuals at baseline (blue line) we see that this corresponds to 15,958 AU/mL, or 2266 BAU/mL. The latter may serve as a more conservative expectation of immunocompetent vaccine response.

With the aim of potentially coalescing an initial vaccine CoP based on the peak binding antibody response observed post-vaccination in primarily immunocompetent individuals, we searched for studies that reported vaccine response measures of binding antibody assays (i.e., S1 or S1-RBD) in BAU/mL. Peak median or mean antibody responses post-vaccination from 10 studies are summarized in Table 1. From this summary, we extracted only IgG S1-RBD measures and excluded viral vector vaccine responses as those appeared to differ from mRNA and recombinant protein vaccine responses. This left five studies that provided a peak IgG S1-RBD range of 1372–2744 BAU/mL post-vaccination [22,27,28,30,31]. We observed that the peak IgG S1-RBD response measured in our healthcare worker cohort, 2266 BAU/mL (Figure 1), for infection-naive individuals at baseline, falls within this range. Since some responses in Table 1 were reported as median values and others as mean values, this range would slightly shift if all were reported as the mean or median. Nevertheless, this range may serve as a suggested initial, conservative, binding antibody CoP for mRNA and recombinant protein vaccines. Such a range may need to be updated on a rolling basis as vaccines are updated and stratified by vaccine type.

### Establishing a Serological Correlate of Protection (CoP) and the Issue of Variants

CoPs for SARS-CoV-2 have not been agreed upon by guideline-forming bodies. Neutralization and other antibody measures correlated to vaccine efficacy have been published in a small group of clinical studies attempting to define CoPs (i.e., COVE, COV002 (AZD1222), ENSEMBLE, PREVENT-19) [22,23,24,25,27]. These four primary vaccine endpoint studies have also been summarized by Gilbert et al. and they proposed that a neutralizing antibody CoP is supported by the data. Both binding and neutralizing antibody response data for these four studies are summarized in the beginning of Table 1. Gilbert et al., also graphed the correlation of neutralization titers to vaccine efficacy from these four studies. We extrapolated from their graph that ~90% vaccine efficacy would correspond to a neutralization titer of ~100–300 IU_50_/mL (with wide error bars) [57]. They go on to acknowledge that threshold CoPs are desirable but likely unattainable for SARS-CoV-2. They also acknowledge that the four primary summarized studies used a clinical endpoint measure of symptomatic COVID-19, and that CoPs may vary with outcome of interest. Prevention of severe COVID-19 is now the most important outcome.

At the time of this publication there are a few neutralization assays that have received FDA EUA, and they are all surrogate assays. One representative example is the cPASS SARS-CoV-2 antibody neutralization test (GenScript), which is a semi-quantitative competitive surrogate neutralization test in an enzyme-linked immunosorbent assay (ELISA) format measuring the binding interaction of ACE2 with RBD (ACE2-RBD). Although ACE2-RBD inhibition assays are only proxies for SARS-CoV-2 neutralization, they afford a degree of convenience over the gold standard PRNTs, which are time consuming, expensive, and require BSL 2 or 3 containment facilities. The drawback of the surrogate assays is that they do not measure the full diversity of virus neutralizing activity that occurs outside of S1-RBD antibodies, nor do they necessarily reflect activity against variants of concern (VOCs) or variants of interest (VOIs). The use of live virus in PRNT assays reveals differential responses to antibodies present in patient samples targeting ancestral strains as well as VoCs [58].

Antibody testing that can be performed in a high throughput manner is key for routine clinical use. Automated binding antibody immunoassays measuring anti-S1-RBD IgG, total antibody, or other specific immunoglobulin subclasses, have been developed by several manufacturers and are progressively being standardized. A primary tool for standardization of SARS-CoV-2 antibody assays are the World Health Organization (WHO) International Standard materials, from which results for binding assays are reported in binding antibody units per milliliter (BAU/mL). The first WHO standard for quantitative measurement of SAR-CoV-2 antibodies was established in December 2020 with a goal to make the results of assays (meeting a defined specificity, e.g., >99.5%) comparable between laboratories and groups using them to measure vaccine response [59,60,61]. A focus on S1-RBD binding antibody measurement makes sense for a CoP because S1 is the key to cell entry and the dominant viral antigen for generation of neutralizing antibody populations. Furthermore, current SARS-CoV-2 mRNA vaccines target S1-RBD, although this may change in the future. Recent additional support for use of an S1 binding antibody measurement comes from Goldblatt et al., who report that assays measuring S1 antibodies (either neutralization or binding) both have predictive value for an accurate CoP, but that S1 binding antibody measurements provide the highest statistical correlation [62,63]. They also measured humoral immune response to four COVID-19 vaccines BNT162b2 (Pfizer), mRNA1273 (Moderna), ChadOx1/AZD1222 (AstraZeneca), or Ad.26COV2.S (Janssen) and observed that anti-S1 IgG binding antibodies (MSD SARS-Coronavirus Panel 7, Rockville MD) were highly correlated with ID50 neutralization when using a validated pseudovirus neutralization assay [64]. The mean protective threshold observed for anti-S1 binding antibody for wild-type (WT) virus was 154 BAU/mL. Of note, the MSD panel 7 multiplex assay used does not have FDA EUA clearance and therefore we could not assess the response of WT spike antigen in their panel to patients infected with different variants. This would be of interest because manufacturers of FDA EUA assays should assess their assay’s response to variants, and most utilize WT S1 or S1-RBD as an antigen at this time. However, the authors attempted to assess the effect of variants on vaccine response because the MSD panel includes spike antigens derived from WT, B.1.1.7 (Alpha variant) and B.1.617.2 (Delta variant) and a decreasing response from WT to Delta was observed for each of four vaccine types with WT having the highest binding antibody response amongst each vaccine group. The lowest responses were among the AstraZeneca and Janssen vaccine recipients, while the Moderna and Pfizer recipients maintained responses greater than or equal to 331 BAU/mL. This is not surprising, given that the first generation of mRNA and adenovirus vaccines made public used S1-RBD derived from WT virus. Nevertheless, the correlation of anti-S1 IgG with vaccine efficacy, even when using antigens from dominant WT, Alpha, and Delta variants, was high with an overall rank correlation of ρ = 0.90.

We propose two approaches to establishing an antibody CoP for clinical use. The more complicated approach could be as follows: (1) select a neutralization antibody assay that may be used as a reference method against which binding antibody assay(s) (e.g., IgG or total antibody against S1-RBD) are correlated, (2) only use clinical grade binding antibody assays that are standardized and reporting in BAU/mL, and (3) correlate the relevant standardized binding assays to the neutralization antibody reference method with acceptable effectiveness rate (e.g., 70–90% effectiveness). This seemingly straightforward process is, however, complicated by the rise of variants, for which such a process might be performed for each pervasive variant. The second, simpler approach to a potential clinical CoP is as follows: Base an antibody CoP threshold or range on the average peak IgG S1-RBD (or other antigenic target) binding antibody response achieved ~4 weeks from vaccination from a meta-analysis of thousands of immunocompetent individuals with ‘protection’ defined as prevention of severe illness requiring hospitalization. We previously discussed the range of peak IgG S1-RBD vaccine responses that can be deemed conservatively adequate in the prior section (i.e., 1372–2744 BAU/mL) and could help define an initial CoP target for mRNA and recombinant protein vaccines.

Moderna and Pfizer second generation bivalent vaccines became available in late summer 2022. On 11 September 2023, the FDA authorized updated COVID-19 vaccines from Pfizer/BioNTech and Moderna that address a recently circulating Omicron subvariant, XBB.1.5, laying the foundation for a fall vaccination campaign [65]. Thus, it could make sense to base a CoP on a rolling evaluation of thousands of immunocompetent individuals using standardized binding, and neutralization assays as that is what would happen for clinical trials of any new vaccines regardless. The binding antibody antigen target can also be updated as vaccines are updated to incorporate other viral antigens (e.g., S1-NTD, S2-FP, and/or S2-SH). Lastly, it should be recognized that CoPs are just that, a correlation, and should not be considered an absolute measure of protection by doctors or patients.

## 3. Established and Developing SARS-CoV-2 Antibody Clinical Use Cases

### 3.1. Diagnosis of Prior Recent Infection

Patients presenting 3 to 4 weeks into illness who may not have consistently detectable viral load may be diagnosed using an IgG test against S and/or N SARS-CoV-2 antigens. However, it is the antibody to N antigen specifically that would indicate prior or recent infection in a vaccinated person as the vaccines available in the U.S. all elicit S1-RBD antibody responses; but measuring antibodies to both antigens may give a better idea of the overall humoral response. It is possible to use a total Ig test for S1-RBD in this case as well [66,67].

### 3.2. Convalescent Plasma (When Therapeutics Will Not Work against Variants)

Identifying convalescent plasma donors with sufficiently high levels of binding and/or neutralizing antibodies for COVID-19 treatment was an active area of research for several months in the early phases of the pandemic and was granted EUA status by the FDA along with some guidelines on identification of appropriate plasma. As of December 2021, only “high titer” plasma, corresponding to approximately 200 BAU/mL IgG S1, as assessed by specific antibody assays, has EUA [68]. However, the urgency of this use has waned as the primary utility lies with early-diagnosed hospitalized patients. Furthermore, positive outcomes, such as prevention of disease progression and death, appear to be limited with this approach [69,70]. With Delta and earlier variants, the use of convalescent plasma appeared to be outmoded by the introduction of vaccines and monoclonal antibody therapeutics (e.g., bamlanivimab plus etesevimab, casirivimab plus imdevimab (REGEN-COV), and sotrovimab). However, bamlanivimab plus etesevimab and casirivimab plus imdevimab were determined to be relatively ineffective against Omicron variant (B.1.1.529) that emerged in late 2021, but sotrovimab retained some efficacy [71]. Monoclonal antibody therapeutic efficacy was further confounded by the emergence and eventual dominance of the Omicron BA.2 subvariant in the first several months of 2022 [72]. Due to the reduced applicability of these therapeutics with Omicron or other subvariants (current and future), some utility for convalescent plasma has resurged for critically ill or susceptible individuals in this case. Further, the evolution of SARS-CoV-2 demonstrates that monoclonal therapeutics for rapidly evolving pathogens will require agility in their development to maintain effectiveness. To this end, the SARS-CoV-2 Assessment of Viral Evolution (SAVE) program published a perspective paper on their international collaborative approach to rapidly identify variants and their impact on immune protection and development of countermeasures (i.e., therapeutics and vaccines). Their aim is to serve as a guidepost for future development of models, diagnostics, and therapeutics against rapidly evolving pathogens [73].

### 3.3. MIS-C

Although rare, diagnosis of suspected multisystem inflammatory syndrome in children (MIS-C) in response to SARS-CoV-2 infection became an important differential for clinicians and pediatricians during the pandemic. To identify MIS-C resulting from SARS-CoV-2 infection, both nucleic acid amplification testing (NAAT) and serology are performed. Many children with this syndrome will have detectable antibodies to SARS-CoV-2 but a negative NAAT test. It has been suggested that the syndrome results from an abnormal immune response to the virus, with some clinical similarities to Kawasaki disease (KD), macrophage activation syndrome (MAS), and cytokine release syndrome. However, based on the available studies, MIS-C appears to have an immunophenotype that is distinct from KD and MAS [74,75]. The exact mechanisms by which SARS-CoV-2 triggers such an abnormal immune response requires further elucidation. A post-infectious process is suggested, based on these cases generally presenting after the COVID peak in various communities [76].

### 3.4. Booster Doses in Immunosuppressed Populations

Currently, it is recognized that antibodies against viral S protein can be measured as a general assessment of response to vaccination (i.e., seroconversion) as the vaccines available in the U.S. and most of the developed world elicit responses to S1-RBD antigen. While the vast majority of healthy individuals (approaching 100%) will mount robust antibody responses to RNA and viral vector vaccines targeting the viral S protein [77], immunosuppressed or compromised individuals (i.e., patients taking immunosuppressants, steroids, or B-cell depleted) typically demonstrate no or low antibody responses in comparison [78,79]. This lack of response has been demonstrated in both solid organ transplant (SOT) and hematologic cancer patients. A study by Monin et al., examined the immunogenicity of the BNT162b2 (Pfizer–BioNTech) vaccine after one and two doses in patients with both solid organ and hematologic cancers [80]. They observed after two doses that 95% (CI 75–99) of solid cancer patients, 100% (CI 76–100) of healthy controls, and 60% (CI 23–88) hematological cancer patients were seropositive. It should be noted that the hematological cancer cohort was small at the two-dose timepoint.

In a study by Marinaki et al., out of 34 SOT patients receiving two doses of the BNT162b2 vaccine 58.8% had positive IgG S1-RBD antibodies detected [81]. This low rate of seropositivity in SOT patients was corroborated by an earlier report by Boyarsky et al., who observed a seroconversion rate of 46%. Furthermore, while a two-dose regimen of RNA vaccine or a single dose of DNA vaccine may not be sufficient for a given immunocompromised or suppressed individual, a third or additional dose may prove the trick. Werbel et al., showed that approximately one-third of SOT patients who were seronegative after completing an initial vaccination series seroconverted after a third dose. Later, Kamar et al., reported a similar rate of seroconversion after a third dose of vaccine in SOT patients with a 28% increase in seroconversion after the third dose for a final overall rate of seroconversion of 68%. They have since further reported on the effectiveness of a fourth dose in boosting antibody responses in SOT patients [82]. Thus, measuring IgG S or IgG S1-RBD antibody levels in these individuals 2–4 weeks after a typical vaccine regimen can help determine whether these patients may benefit from additional doses.

### 3.5. Routine Booster Dosing, Updated Vaccines, and General Population Antibody Response

We have now twice observed vaccines updated to address variants from Pfizer and Moderna in 2022 and 2023, with others pending. And we can likely expect to see these updated further to address new variants both for initial population-based vaccination as well as booster dosing [65,83,84]. As expected, the Pfizer–BioNTech and Moderna bivalent vaccines authorized in August 2022 showed enhanced neutralizing antibody response to Omicron variants, while also inducing immune response to the original (ancestral) SARS-CoV-2 strain. Monovalent (ancestral) vaccines are now no longer available in the U.S.

Equity of vaccine availability in the U.S. has not been a sustained issue; however, this has not panned out globally and low-income countries were particularly affected during the height of the pandemic. The argument was previously made that the pandemic will not end until global vaccine equity is achieved. As of 6 September 2023, 76.89% of people in high-income countries have received at least one dose of vaccine, vs 35.58% in low-income countries [85]. At this point, the reasons for this disparity may be multi-factorial with the downgrade of the pandemic to an endemic. However, considering “low responders” to vaccine who may require many doses, waning protection in all populations over time, and continued equity issues, the case for serological testing prior to administering primary or additional vaccine doses could be warranted in many regions of the world. 

Furthermore, some individuals may prefer not to receive primary or additional doses if their antibody response can first be measured and deemed adequate. A recent survey examining low-uptake of SARS-CoV-2 vaccine booster doses among 2196 adults in the Arizona CoVHort showed the most commonly reported reason for not receiving a booster dose was a prior SARS-CoV-2 infection (39.5%), followed by concern about vaccine side effects (31.5%), believing that the booster would not provide additional protection over doses already received (28.6%), concern about booster safety (23.4%), and/or that it would not protect from SARS-CoV-2 infection (23.1%) [86]. Additionally, a global survey of 23 countries with 23,000 respondents conducted in summer 2022 showed that overall vaccine hesitancy was ~21% [87]. Natural exposure to viral variants has, of course, continued in both the vaccinated and unvaccinated such that almost every immunocompetent person may now be expected to have some degree of measurable antibody response. Without an agreed-upon CoP, measurement of antibody levels can still provide clinical utility through comparison of an individual’s peak S1-RBD antibody response, which occurs 2–4 weeks after infection or vaccination, to what is observed in immunocompetent individuals [88,89]. We previously discussed what may be deemed an adequate response in Section 2.

Clinical laboratories can help educate healthcare providers on what a typical peak response would be for a given assay as assay standardization and a consensus CoP are not ubiquitous.

## 4. Limitations of Serology Testing

This section highlights some observed and foreseen limitations. Previously discussed use cases (including some additional use cases) and known limitations are summarized in Table 2.

### 4.1. Timing and Lack of Standardization

The case against using SARS-CoV-2 antibody testing, at least in a routine manner, is as follows: (1) The majority of individuals, approaching 100% in relatively healthy populations, will seroconvert following vaccination. Quantifying specific SARS-CoV-2 antibody responses to immune protection is an ongoing area of study. (2) Many available SARS-CoV-2 serology tests are not yet standardized, although this could now readily be addressed by employing WHO international standards. (3) Timing of serological testing is key, i.e., it should not occur sooner than two weeks, but preferably four weeks after vaccination or diagnosis to optimize sensitivity and specificity.

Additionally, many patients and providers may not understand what the results mean. One test may be reported in AU/mL, another μg/mL, and yet others in BAU/mL (i.e., WHO standardized) and those values will certainly not be interchangeable. Further, many providers may still not understand the difference between using an assay that measures N antibodies and one that measures S antibodies (or subunits of S); nevertheless, clinical labs are doing their part to highlight these differences in utility. Additionally, as stated before, what constitutes an adequate level of binding antibody, with a primary focus on S1-RBD antigen, needs consensus.

FDA EUA status was granted for over 80 antibody tests at the time of this publication; however, only a small subset is in common clinical usage [90]. The clinical community and assay manufacturers need to push further towards broad standardization so that results can become interchangeable and mean the same thing to clinicians and patients. This is happening but remains a movement in progress.

### 4.2. Impact of Variants on the Antibody Response

Earlier observations show that the neutralizing antibody response induced by mRNA vaccination, prior to bivalent versions, is approximately 3-fold lower when individuals are infected by Omicron variants BA.4 and BA.5 as compared with earlier variants BA.1 and BA.2 [91].

That said, as required by the FDA in a letter dated September 2021, manufacturers of certain molecular, antigen and serology tests are now required to routinely monitor emerging viral mutations and their potential impact on the performance of the authorized SARS-CoV-2 test(s). As examples, Roche and Abbott are manufacturers are of highly utilized SARS-CoV-2 binding antibody assays and have evaluated the impact of variants, from Alpha to Omicron, and both report they have not observed a reduced capability to detect binding antibodies induced by these VOCs [92,93].

Due to incomplete assay standardization and need for additional CoP studies, the FDA and CDC have been hesitant to directly state that SARS-CoV-2 antibody tests (i.e., neutralization or binding) can be used to monitor vaccine effectiveness. However, this could be changed with an understanding of the issues discussed herein and a broader analysis of studies performed on longitudinal antibody response post-vaccination, which we have reviewed and summarized. We believe that additional vaccine response studies looking at the endpoint measure of prevention of severe disease and using only standardized antibody assays are needed.

### 4.3. A Consideration of the Cellular Response

The immune response to vaccination is less nuanced than the immune response to natural infection where exposure to whole virus and multiple protein antigens is involved; and the T cell and B cell populations generated will also be broader. Further, we do not know whether low or “negative” antibodies but high or detectable virus-specific T cell populations, or simply the presence of memory T cells in all populations could provide a measure of protection against severe infection, particularly in the long term [50,94,95]. It is likely that the importance of the T cell response in preventing severe disease (i.e., leading to hospitalization and death) from SARS-CoV-2 has been underestimated thus far [96].

Although this work serves primarily as a review of clinical utility of serology assays for SARS-CoV-2, we will briefly address some of the latest developments in clinical measurement of the T cell response. In June 2022, Schwarz et al., published two variations of a method for measurement of T cell activation on quantitative polymerase chain reaction (PCR) [97]. Both methods measure CXCL10 messenger ribonucleic acid (RNA), which is a chemokine strongly associated with activation of antigen-specific T cells. The methods allow for faster-than-typical measurement of the T cell response at 24 h, and the authors discuss its scalability for measuring long-term vaccine response. This is undoubtedly an important method to aid in the ongoing effort to assess vaccine durability and protection in the academic community. However, the scalability, cost, and timeframes desired for high throughput analysis can still be prohibitive to such a method the clinical setting, especially if the intention is to measure functional T cell immunity to SARS-CoV-2 for revaccination strategies in vulnerable populations. Furthermore, for long-term analysis, the persistence of memory B cells are likely critical to prevention of infection and/or mild infection courses as it has already been observed that the humoral response is essential to SARS-CoV-2 viral clearance and immunity [51,98,99]. Also, within the first 8 months after infection in immunocompetent individuals, it has been observed that antibodies and T cell responses decline, while the memory B cell response actually increases [100]. Nevertheless, a faster antigen-specific T cell measurement method that does not require isolation of buffy coat, flow cytometry, or large volumes of blood is indeed an advancement in the ongoing efforts to assess the extent and durability of the immune responses to SARS-CoV-2. It would not be surprising to observe that antigen-specific T cells to SARS-CoV-2 persist for years, if not decades, as was observed with SARS-CoV-1 [50].

## 5. Conclusions

This review examines existing evidence supporting use of SARS-CoV-2 antibody testing in the clinical setting. Although substantive information about the host immune response to SARS-CoV-2 infection has been published, the accumulated evidence has not yet translated into FDA or CDC endorsement of SARS-CoV-2 antibody testing to monitor either vaccine effectiveness, or to inform CoPs against severe SARS-CoV-2 infection. A key reason is that antibody assay standardization is not yet ubiquitous, and there is a need for even more clinical studies that utilize such standardized assays to define a practical CoP or set of CoPs based on vaccine format (e.g., mRNA, viral vector, etc.). Additionally, more studies evaluating binding antibody testing as a proxy for measurement of neutralizing antibodies and, in turn, susceptibility to infection are warranted, especially as vaccines are updated in the future.

Clinical studies and research have attempted to define such correlates by assay and vaccine type, as was summarized in Table 1. Additionally, several clinical use cases were highlighted that have already proven useful, e.g., MIS-C, convalescent plasma, booster vaccine equity, etc. Furthermore, most arguments against clinical use of serology testing are ameliorated by appropriate education of clinicians regarding antigen targets, timing of testing, and understanding of reporting units.

It was further discussed that a conservatively high target for a CoP (both for relevant neutralizing and binding antibody assays) could now be considered, one that is revised based on effectiveness data gathered over time. Also, a simpler way to define a CoP, based on extracting the average peak binding and/or neutralizing antibody response to vaccines in immunocompetent populations, may now be defined and updated overtime. This peak, immunocompetent response can also serve as a potential target for booster dosing in the immunocompromised and elderly. An initial CoP for mRNA and recombinant protein vaccine approaches was extracted for the peak S1-RBD response as follows: 1372–2744 BAU/mL from published studies reporting in standardized units. This target would need to be confirmed and potentially adjusted for updated booster vaccines. The studies summarized in Table 1 also reflect that binding antibody CoP targets would likely need to be defined separately for other vaccine formats (i.e., viral vector approaches).

Lastly, it is important to define the end goal for a measure of vaccine effectiveness and thus the corresponding CoP. Is it protection against severe disease requiring hospitalization or simply infection? Early in the pandemic, vaccine effectiveness was based on prevention of symptomatic infection, but we now know that the rise of variants and subvariants should likely change the endpoint measure to severe disease requiring hospitalization, in which case CoPs may be much more readily defined and updated.

## Figures and Tables

**Table 1:** In bold are antibody responses that were used to determine a broad, conservative range of peak S1 and S1-RBD IgG binding antibody values post-vaccination that may indicate a potential binding antibody CoP target, 1372–3803 BAU/mL, in largely immunocompetent populations and a potential booster target range for the immunocompromised and elderly. However, the high end of this range is from two studies using S1 IgG, rather than S1-RBD IgG, and it is likely that the higher values observed are in part due to more measurable antibodies against the entire S1 subunit than S1-RBD alone. If we only focus on S1-RBD IgG values the CoP range narrows to 1372–2744 BAU/mL and covers both mRNA vaccine (i.e., Pfizer and Moderna) and recombinant protein vaccine (i.e., Novavax) approaches. Additionally, some binding antibody responses were reported as median values and others as mean values, so the S1-RBD IgG range indicated would slightly shift if all were mean or median values. Oxford/AstraZeneca (AZD1222), Janssen, and Sputnik viral vector vaccine responses were excluded as they appear to produce lower responses than mRNA vaccines. Peak antibody responses post-vaccination series (i.e., 1 or 2 doses as specified by manufacturer) in largely immunocompetent populations extracted from clinical trials and research studies.

Antibody Assay (Source)	N (Received Vaccine)	Antibody Level or Titer	Study	Vaccine	Doses	Percent Efficacy	Clinical Endpoint Measure	Days Post Second Dose	Dominant Variant at Time of Study	Population Studied
Anti-spike IgG (MSD Diagnostics)	1051	1890 BAU/mL ^†^ GMC ^Ω^ (95% CI: 1499 to 2465)	COVE [22]	mRNA-1273 (Moderna)	2	90%	Symptomatic COVID-19	Median 28	Alpha	General; 47% female, 34% > 65 y/o, 40% at high risk for severe COVID-19, 54% non-white. Infection naive at baseline
**Anti-S1RBD IgG** **(MSD Diagnostics)**		**2744 BAU/mL** **^†^** **GMC** **^Ω^** **(95% CI: 2056** **to 3664)**								
Pseudovirus Neutralization assay		160 IU_50_/mL ^∞^ GMC ^Ω^ [95% CI: 170 to 220]								
Anti-spike IgG (MSD Diagnostics)	1155	264 BAU/mL ^†^ GMC ^Ω^ (95% CI: 108 to 806)	COV002 [23]	ChAdOx1 nCoV-19 (AZD1222)	2	80%	Primary symptomatic COVID-19	28	Alpha	General; 57.9% female, 74.1% < 55 y/o, 24.9% at high risk for severe COVID-19, 92.3% white. Infection naive at baseline
Anti-S1RBD IgG (MSD Diagnostics)	1155	506 BAU/mL ^†^ Median (95% CI: 135 to NC ^‡^ [beyond data range])								
Pseudovirus Neutralization antibodies	828	26 IU_50_/mL GMC ^Ω^ (95% CI: NC ^‡^ to NC ^‡^)								
Live-Virus Neutralization antibodies	412	247 normalized neutralization titer (NF_50_) (95% CI: 101 to NC ^‡^)								
Anti-spike IgG (MSD Diagnostics)	826	238 BAU/mL 97.5th percentile	ENSEMBLE [25]	Ad26.COV2.S (Janssen)	1	89%	Moderate to severe-critical COVID-19	29	Alpha	General; 44.8% female, 50.4% ≥ 60 y/o, 51.7% at high risk for severe COVID-19, 49.3% non-white. Infection naive at baseline
Anti-S1RBD IgG (MSD Diagnostics)		173 BAU/mL 97.5th percentile								
Pseudovirus Neutralization Assay		96.3 IU_50_/mL ^∞^ 97.5th percentile								
Anti-spike IgG (MSD Diagnostics) **Anti-S1RBD IgG** **(MSD Diagnostics)**	19,996	1552 BAU/mL ^†^ GMC ^Ω^ (95% CI: 1407 to 1713) **2123 BAU/mL ^†^** **GMC ^Ω^** **(95% CI: 1904 to 2369)**	PREVENT-19 [24,26,27]	NVX-CoV2373 (Novavax)	2	87.7%	Symptomatic COVID-19	35	Primarily Alpha	General; 46.7% female, 46.7% ≥ 65 y/o, 49.7% at high risk for severe COVID-19, 42.5% non-white. Infection naive at baseline
Pseudovirus Neutralization Assay		461 IU_50_/mL ^∞^ GMC ^Ω^ (95% CI: 404 to 526)								
**Anti-S1RBD IgG** **(Abbott Diagnostics)**	52	**2018.0 BAU/mL** **^†^** **Median**	Deeba et al., 2022 [28]	BNT162b2 (Pfizer)	2	N/A	N/A	~21	Omicron (BA.2)	Healthcare professionals in Cypress
	45	182.1 BAU/mL Median		ChAdOx1 nCoV-19 (AZD1222)	2					
**Anti-S1RBD IgG** **(In-house ELISA)**	86	1209 BAU/mL ^†^ Mean (in previously infected) or >694 BAU/mL ^†^ (correlated to excellent neutralizing activity)	Claro et al., 2022 [29]	Sputnik	2	91% in infection naive; 100% in previously infected	Good to excellent neutralizing activity as defined by WHO standardized neutralizing antibody response of 100–400 or greater IU/mL	42	Alpha (B.1.17)	Healthcare professionals in Venezuela
**Anti-S1RBD IgG** **(Abbott Diagnostics)**	1343	**1432 BAU/mL** **^†^** **Median**	Bordi et al., 2022 [30]	BNT162b2 (Pfizer)	2	N/A	N/A	~30	Delta	Healthcare professionals in Italy
**Anti-S1RBD IgG (Snibe Co., MAGLUMI)**	2248	**1372 BAU/mL** **^†^** **Median**	Lo Sasso et al., 2021 [31]	BNT162b2 (Pfizer)	2	N/A	N/A	10–20	Mainly Alpha	Outpatients presenting for blood draw in Italy
**Anti-S1 IgG** **(Euroimmun Anti-SARS-CoV-2-QuantiVac-ELISA)**	93 (age < 60 y/o)	**3702 BAU/mL** **^†^** **Mean**	Muller et al., 2021 [32]	BNT162b2 (Pfizer)	2			17	Mainly Alpha	Infection naive adults (younger and older elderly populations) from nursing home facilities in Germany
	83 (age > 80 y/o)	1332 BAU/mL ^†^ Mean								
In-house PRNT (neutralization) assay	93 (age < 60 y/o)	ID50 ∗: 97.8%								
	83 (age > 80 y/o)	ID50 ∗: 68.7%								
**Anti-S1 IgG** **(Euroimmun Anti-SARS-CoV-2-QuantiVac-ELISA)**	107 out of 263	2478 BAU/mL ^†^ Mean (before Omicron BA.1/2 breakthrough)	Kajanova et al., 2023 [33]	Several vaccines	At least 2 (or series completed)		No breakthrough infection during period studied	N/A	Omicron BA.1/2	Healthy adult volunteers; 76% female, 24% male; median age 45 y/o, in Slovakia with previous vaccination series completed
	152 out of 263	**3803 BAU/mL** **^†^** **Mean** **(before NO Omicron BA.1/2 breakthrough)**				100%				
	141 out of 263	>6201.5 BAU/mL ^†^ (upper most quartile of responses most likely to avoid breakthrough infection)								

† BAU/mL = binding antibody units/milliliter (WHO standardized); Ω GMC = geometric mean concentration; ∞ IU_50_/mL = concentration at which 50% neutralization is observed expressed in international units (IU) per milliliter.; ‡ NC = not computed; ∗ ID50 = inhibitory dilution at which 50% neutralization is observed.

**Table 2:** Clinical Use Cases and Limitations for Measurement of SARS-CoV-2 Antibody Levels.

Clinical Use Cases	
Late Diagnosis	Patients presenting 3–4 weeks after symptom onset with negative viral testing can have recent exposure/infection confirmed with appropriate antibody testing (e.g., IgG S and IgG N).
Convalescent Plasma (CP)	Screening CP donors for appropriately high levels of antibodies prior to donation.
Multisystem inflammatory syndrome in children (MIS-C)	Many children with MIS-C following infection will have detectable antibodies to SARS-CoV-2 but a negative NAAT test.
Correlates of protection (CoPs)	A conservatively high antibody response, ideally standardized to WHO or other acceptable standardization (i.e., reportable in BAU/mL) for IgG or total antibody to S1-RBD could be proposed based on correlation to neutralizing antibody assay(s) or based on vaccine response achieved in immunocompetent populations. Further the endpoint for protection needs to be agreed upon (e.g., protection from severe disease requiring hospitalization).
Post-acute Sequelae of COVID-19 (PASC)	It may be useful to follow antibody levels pre- and post-vaccination in these patients to understand their individual responses, including neutralizing antibody development.
Booster vaccine dosing	Antibody response can be used as a general determinant for booster dose necessity where vaccine availability is limited (e.g., in the developing world) or a patient’s desire to not be exposed to additional, potentially unnecessary doses (e.g., those who have experienced non-life-threatening side effects).
**Limitations**	
Negative results in the acute stage post-infection or post-vaccine	Need to wait at least 14 days post-symptomatic infection to assess antibody levels. To assess peak antibody response, waiting 3–4 weeks post-vaccine dose or post-infection is suggested.
False positives	Despite low sequence identity (i.e., ~30%) for S protein between SARS-CoV-2 and other alpha and beta coronaviruses (excluding SARS-CoV-1), positive antibody reactions from recent exposure to other coronaviruses (e.g., OC43 and HKU1) may occur.
Binding antibody correlates of protection (CoPs)	An agreed-upon conservative CoP threshold or range remains to be confirmed by guideline-forming bodies and will likely need to be updated over time. A conservative range of peak IgG S1-RBD responses (i.e., 1372–2744 BAU/mL) applicable to the first-generation mRNA and recombinant protein vaccines was extracted from several studies (see Section 2).
Waning of antibodies	It has been noted by many studies that antibody levels wane over time. The timing for a booster dose has been suggested by the CDC based on age, immunocompromised status, and type of vaccine administered. At the low end, a period of ~3–6 months for boosting may be suggested for the immunocompromised or elderly. Additionally, a peak antibody response may be expected at the 3–4 week timeframe for most individuals and is the suggested ideal time to measure antibody response.

## Data Availability

Requests for de-identified data may be directed to the corresponding authors (KS and SC) and will be reviewed by the Office of Research Administration at Cedars-Sinai Medical Center prior to issuance of data sharing agreements, which are designed to ensure patient and participant confidentiality.
